# Gold-seeded Lithium Niobate Nanoparticles: Influence of Gold Surface Coverage on Second Harmonic Properties

**DOI:** 10.3390/nano11040950

**Published:** 2021-04-08

**Authors:** Rachael Taitt, Mathias Urbain, Zacharie Behel, Ana-María Pablo-Sainz-Ezquerra, Iryna Kandybka, Eloïse Millet, Nicolas Martinez-Rodriguez, Christelle Yeromonahos, Sandrine Beauquis, Ronan Le Dantec, Yannick Mugnier, Pierre-François Brevet, Yann Chevolot, Virginie Monnier

**Affiliations:** 1 Univ Lyon, ECL, INSA Lyon, CNRS, UCBL, CPE Lyon, INL, UMR5270, 69130 Ecully, France; rachael.taitt@ec-lyon.fr (R.T.); ana-maria.pablo-sainz-ezquerra@ec-lyon.fr (A.-M.P.-S.-E.); iryna.kandybka@master.ec-lyon.fr (I.K.); eloise69210.millet@gmail.com (E.M.); nicolasmaro93@gmail.com (N.M.-R.); christelle.yeromonahos@ec-lyon.fr (C.Y.); yann.chevolot@ec-lyon.fr (Y.C.); 2 Univ. Savoie Mont Blanc, SYMME, F-74000 Annecy, France; mathias.urbain45@gmail.com (M.U.); Sandrine.Beauquis@univ-smb.fr (S.B.); Ronan.le-Dantec@univ-smb.fr (R.L.D.); Yannick.Mugnier@univ-smb.fr (Y.M.); 3 Université de Lyon, Université Claude Bernard Lyon 1, UMR CNRS 5306, Institut Lumière Matière, F-69622 Villeurbanne, France; zacharie.behel@univ-lyon1.fr (Z.B.); pfbrevet@univ-lyon1.fr (P.-F.B.)

**Keywords:** lithium niobate, gold seeds, plasmon, second harmonic generation, surface, hyperpolarizability

## Abstract

Hybrid nanoparticles composed of an efficient nonlinear optical core and a gold shell can enhance and tune the nonlinear optical emission thanks to the plasmonic effect. However the influence of an incomplete gold shell, i.e., isolated gold nano-islands, is still not well studied. Here LiNbO_3_ (LN) core nanoparticles of 45 nm were coated with various densities of gold nano-seeds (AuSeeds). As both LN and AuSeeds bear negative surface charge, a positively-charged polymer was first coated onto LN. The number of polymer chains per LN was evaluated at 1210 by XPS and confirmed by fluorescence titration. Then, the surface coverage percentage of AuSeeds onto LN was estimated to a maximum of 30% using ICP-AES. The addition of AuSeeds was also accompanied with surface charge reversal, the negative charge increasing with the higher amount of AuSeeds. Finally, the first hyperpolarizability decreased with the increase of AuSeeds density while depolarization values for Au-seeded LN were close to the one of bare LN, showing a predominance of the second harmonic volumic contribution.

## 1. Introduction

The synthesis of functional, multi-material nanoparticles (NPs) has been of particular interest for over two decades, as it allows one to bring together or to enhance various physicochemical properties. Several types of multi-material NPs with many different architectures and geometries have been reported such as Janus [1], core-shell [2] or hollow [3] NPs. Gold-seeded NPs, i.e., NPs decorated with a non-continuous shell composed of gold nano-islands, have found application in various fields such as catalysis [4], sensing [5] or enhanced Raman scattering [6]. The core material of these gold-seeded NPs includes silica [7], iron oxide [6], titanate and other oxides [8] and even polystyrene [9] or liposomes [10]. The present report focuses on the synthesis of lithium niobate core NPs decorated with gold seeds.

Lithium niobate (LiNbO_3_) is a well-known mixed-metal oxide exhibiting multifunctional properties that have long been optimized according to its content of intrinsic and extrinsic defects [11]. Among them, the piezoelectric, electro-optic and nonlinear optical properties are all due to its non-centrosymmetric crystalline structure. Interestingly, the absence of phase-matching conditions at the nanoscale for the so-called harmonic NPs, paved the way to new bio-imaging [12] and photo-triggering [13] approaches when LN NPs, for instance, are used as exogenous biomarkers. In terms of measurable nonlinear optical signals, a very rich multi-order response has recently been measured with the simultaneous detection of ten distinct emissions spanning from the deep ultraviolet to the short-wave infrared region [14]. At the second order of the susceptibility development, namely for the second harmonic generation (SHG) process, the relatively high orientation-averaged second-order susceptibility 〈$\chi^{\left(2\right)}$〉 of LN was previously found to increase from ~10 pm/V [12] to 24 pm/V [15] when the size and shape polydispersity are reduced and for a 1064 nm excitation wavelength. Note that this latter value obtained with LN suspensions of hydrodynamic diameter centered at ~100 nm is very similar to the expected bulk response [15].

Coupling efficient nonlinear optical NPs with gold nanostructures was investigated by several research groups during the last few years, both with NPs deposited on substrates such as nanoantennas [16,17] or on core-shell NPs dispersed in a liquid [18,19,20,21,22]. Concerning core-shell NPs and nanowires, an enhancement of SHG intensities was observed for potassium niobate nanowires coated with a gold shell, at fundamental excitation wavelengths matching the localized surface plasmon resonance of the gold-covered nanowire between 900 and 1000 nm [20]. In another work, it was demonstrated that barium titanate NPs coated with a gold shell exhibit local- and far-field radiative enhancements at different frequencies and the possibility to generate tunable emission in the infrared [23]. In both cases, the nonlinear optical core was coated with a complete gold shell, either rough or smooth. However, the influence of lower gold densities on the resulting SHG properties, i.e., when gold NPs are not closely packed, was never studied to our knowledge. As recently mentioned, the interactions between a large assembly of individual plasmonic NPs are complex and still weakly understood [10,24]. Several plasmonic regimes were identified on gold-coated liposomes depending on the gold surface coverage. In particular, it was found that plasmonic interactions are only effective if gold NPs are sufficiently close or overlap. Additionally, in a recent work, it was shown that the gold NPs arrangements on a silica core influenced the optical response with a red-shifted plasmon band when moving from random to raspberry-like and to bracelet-like gold shell distributions [25].

Here we present the preparation and characterization of lithium niobate core NPs coated with various densities of gold nano-seeds. The gold seeds surface coverage is governed by several parameters including zeta potential values of the core and of the seeds, pH of the NPs dispersion medium, seeds concentration and seeding duration of the core surface. The chemical properties of the core also play a key role and several chemical modifications have been reported. For a silica core, aminosilanes [7] are typically required for the adsorption of gold species. Polyelectrolytes [6] or other positively-charged molecules such as CTAB [26] can also be employed. Other methods involve deposition-precipitation, without prior chemical modification, in presence of a reducer such as urea [8] although it is not efficient for oxides with an isoelectric point lower than 5. The LN core was here modified using a positively-charged polymer, Branched PolyEthylenImine (BPEI) as intermediate linker. PolyEthyleneImine (PEI) and its variants are already widely used in biology. The high cationic charge makes them suitable for complexation with nucleic acids [27] while the high reactivity of the amino groups ensures a wide range of possible functionalization and targeting [28]. BPEI has been used successfully to coat metal oxide NPs [6,29]. In addition, the presence of amino groups has been shown to promote the attachment of small, negatively charged, spherical gold NPs to larger metal oxide NPs and, depending on the molecular weight (MW) of BPEI with respect to the core size, the colloidal stability of these coated NPs can be improved [30]. The surface properties of the BPEI-coated LN NPs were investigated with fluorescamine-based titration and X-ray photoelectron spectroscopy (XPS). Gold-seeded LN NPs were characterized using transmission electron microscopy (TEM), inductive-coupled plasma atomic emission spectroscopy (ICP-AES) and dynamic light scattering (DLS). Hyper Rayleigh scattering (HRS) was then used to measure the hyperpolarizability and depolarization values according to the gold surface coverage.

## 2. Materials and Methods

### 2.1. Materials

Lithium niobium ethoxide (LiNb(OEt)_6_), (99+% metal basis, 5% *w*/*v* in ethanol) was obtained from Alfa Aesar (Kandel, Germany), Teflon cup model number 4749 was obtained from Parr Instrument (Moline, IL, USA), and Nalgene centrifugation tubes were obtained from ThermoFisher (Waltham, MA, USA). Butane-1,4-diol (99%), ethanol, sodium hydroxide (NaOH) pellets, tetrakis(hydroxymethyl)phosphonium chloride (THPC) solution (80% *w*/*v* in water), polyethylenimine branched (BPEI), ∼25,000 g/mol, gold(III) chloride trihydrate (HAuCl_4_.3H_2_O, ≥99.9%, trace metal basis), sodium tetraborate, fluorescamine and hydroxylamine hydrochloride (NH_2_OH, HCl) were all obtained from Sigma Aldrich (Saint-Louis, MO, USA). The HAuCl_4_∙3H_2_O was dissolved in deionized water to give a concentration of 313 mM, stored in a dark bottle and kept in the dark until needed. Unless otherwise stated, any water used was deionized water (18.2 MΩ·cm).

### 2.2. Synthesis of LiNbO_3_ Nanoparticles (LN NPs)

The LN NPs were synthesized by a solvothermal process. Inside an autoclave equipped with a 23 mL Teflon cup, 2.25 mL of lithium niobium ethoxide and 1.25 mL of butane-1,4-diol were added. The mixture was then heated in an oven (Memmert, Schwabach, Germany) increasing the temperature by 5 °C intervals every ten minutes until the temperature of 230 °C was attained, and this temperature was maintained for three days. After cooling to room temperature, the precipitate was transferred to Nalgene centrifuge tubes. Three centrifugation rounds at 13,500 rpm for three minutes were carried out to collect the powder which was washed with ethanol and sonicated for ten minutes. The final LN NPs were dried at 75 °C. They were then dispersed in deionized water at a mass concentration of 1 g/L.

### 2.3. Polymer Adsorption on LN NPs

BPEI polymer was used to coat the LN NPs. Firstly, 5 mg of BPEI was weighed into a glass reactor vessel and dissolved in 5 mL of deionized water while heating at 90 °C. The temperature was maintained at 90 °C with continuous stirring and 0.5 mL of the 1 g/L LN NP dispersion was added to the reactor and left for 4 h. The mixture was allowed to cool to room temperature and then the resulting BPEI coated LN NPs (LN@BPEI NPs) were separated from any BPEI excess by performing six, 10-minute centrifugation washing steps at 10 °C and 9103 g. The LN@BPEI NPs were re-dispersed in deionized water to give a final concentration of 0.35 g/L.

### 2.4. Preparation of Au-Decorated LN NPs (LN@BPEI@AuSeeds NPs)

The preparation of LN@BPEI@AuSeeds NPs was achieved in a two-step process. Firstly, gold seeds (AuSeeds) were synthesized using the protocol reported by Duff et al. [31]. In brief, 540 µL of 0.1 M NaOH was added to 45.5 µL of deionized water followed by 2.14 µL of a 4.2 mM solution of THPC. While stirring, 360 µL of 25 mM HAuCl_4_∙3H_2_O solution was added to the mixture and the stirring was continued for ten minutes, with the mixture undergoing a color change from colorless to brown-red in under 30 s. The AuSeeds dispersion was stored at 4 °C and used for further procedures up to two months post synthesis. To attach the AuSeeds to LN@BPEI NPs, the following theoretical ratios of AuSeeds to LN NPs were tested: 100:1, 300:1, 700:1, 1000:1, 2000:1 and 3000:1. To do so, a series of dilutions of the AuSeeds dispersion with water was completed (given in Table 1), with a final volume of 10.588 mL in each case, and placed in a 50 mL falcon tube.

Then, under stirring, 1 mL of LN@BPEI NPs (0.35 g/L or 1.58 × 1015 NPs/L) was added to the AuSeeds dispersions and allowed to mix for 30 min. The NP dispersion was then kept for 24 h at 4 °C, after which, two 10 min rounds of centrifugation at 10 °C and 9103 g were done to remove the unattached AuSeeds. The resulting LN@BPEI@AuSeeds NPs were re-dispersed in 1 mL of deionized water to obtain again a final concentration of 0.35 g/L.

### 2.5. Fluorescence Spectroscopy for BPEI Concentration Determination

Standard BPEI solutions of concentration 0.05, 0.1, 0.25, 0.5 and 1 g/L were prepared in water. 100 µL of each BPEI solution was added to 2.9 mL of a 0.05 M sodium tetraborate buffer solution at pH 9. Then, 1 mL of 0.3 g/L fluorescamine in acetone solution was added to the buffered BPEI solution and the mixture was allowed to react for 12 h in the dark. Finally, fluorescence measurements of the solutions were performed using a FLS920 spectrofluorimeter (Edinburgh Instruments, Livingston, UK). The fluorescence intensities at the maximum emission of 472 nm (excitation at 388 nm) were extracted and used to plot a concentration calibration curve for BPEI. Then titration of BPEI in supernatants resulting from LN@BPEI centrifugation at each of the 6 washing steps was achieved, analyzing 100 µL of the supernatant after each washing step with the procedure detailed above.

### 2.6. X-ray Photoelectron Spectroscopy (XPS)

XPS measurements were performed with a VSW spectrometer (Dresden, Germany) equipped with a monochromatic X-ray source (Al Kα 1486.6 eV) in which the angle between the incident beam and the detector was the magic angle. The angular resolution was 3° and the take-off angle was 90° relative to the substrate surface. The energetic resolution was 0.2 eV. The data analysis was performed with CasaXPS software (Version 2.3.14). C1s binding energy was set at 285 eV. A Shirley background was subtracted on Si2p and O1s spectra when coming from bulk elements while a linear background was subtracted on C1s and N1s spectra as surface elements. Peaks were fitted by a Gauss-Lorentz curve. Quantification was performed using the Scofield sensitive factors in CasaXPS software.

### 2.7. Hyper Rayleigh Scattering (HRS) Measurements

The SHG properties of LN NPs as well as LN@BPEI@AuSeeds NPs were evaluated using a Hyper Rayleigh scattering (HRS) method with the experimental set-up given in Figure S1. A femtosecond pulsed laser, operating at a wavelength of 800 nm, with a repetition rate of 80 MHz pulses and delivering pulses of 140 fs duration was used. The input polarization angle of the linearly polarized beam was adjusted using a half-wave plate before being focused onto the sample with the use of a 10× microscope objective lens. From a 0.5 × 0.5 cm^2^ quartz cell containing 1 mL of the sample dispersion, the scattered harmonic light was collected at a 90° angle, passing through the analyzer. A photomultiplier tube working in the photon counting regime was used to record the harmonic photons at the output of a spectrometer for wavelength selection.

### 2.8. Other Characterization Techniques

Transmission Electron Microscopy (TEM) was performed with a 2100HT microscope, (JEOL, Tokyo, Japan) equipped with a LaB_6_ electron source, at a 200 kV operating voltage. The microscope was also equipped with a bottom mount Orius SC1000 CCD camera (Gatan, Pleasanton, CA, USA). To prepare the nanoparticle samples for imaging, a carbon-coated 400 mesh copper grid from TED PELLA Inc (Redding, CA, USA), underwent UV-ozone etching at 25 °C for 30 min. Then, 2 µL of nanoparticle dispersion with a concentration between 0.03 and 0.06 g/L for LN and hybrid LN nanoparticles, and 0.035 g/L for the pure AuSeeds dispersions, was dropped onto the grid and allowed drying first in air and then in an oven at 50 °C for 10 min. All of the TEM images were analyzed with the ImageJ software (Version 1.50i). Energy-Dispersive X-ray Spectroscopy (EDS) analysis was performed with the JEOL-2100HT microscope equipped with an X-Max 80 mm^2^ EDS silicon drift detector (Oxford Instruments, Abingdon-on-Thames, UK). The microscope’s operating voltage was set at 200 kV. The EDS spectra were obtained using spot sizes of 35, 25 and 7 nm. The EDS data were analyzed by AZtec software from Oxford Instruments. X-ray Diffraction was performed using Co Kα radiation (λ = 1.789 Å) from an INEL CPS 120 diffractometer. Inductive Coupled Plasma Atomic Emission Spectroscopy (ICP-AES) analysis was performed with an ICAP 6300 and an ICP-AES ICAP 6500 analyzers by ThermoFisher Scientific. The samples were digested in a solution comprised of 4% H_2_SO_4_, 4% HNO_3_ and 4% HCl. HCl was used for samples containing gold. The UV-visible absorption spectroscopy was performed with a SAFAS-UV mc2, double beam spectrometer (Monaco). The spectra acquisition was done by scanning the wavelengths from 400 to 1000 nm, using a bandwidth of 2 nm and a wavelength step of 1 nm. The samples were placed in a quartz cell with 1.0 cm path length. A nanoZS zetasizer (Malvern, UK) was used to obtain the hydrodynamic diameter and zeta potential of the NP dispersions. NPs were dispersed in water at 25 °C and concentrations were kept between 0.03 and 0.06 g/L for LN and hybrid LN nanoparticles, and at 0.035 g/L for the pure AuSeeds dispersions. All measurements were done at a back-scattering angle of 173° with DTS1070 disposable folded capillary cells (Malvern, UK). Zeta potential data were fitted by the Smoluchowski model. The hydrodynamic diameters and zeta potential values reported were the average of triplicate measurements.

## 3. Results and Discussion

### 3.1. Synthesis and Characterization of LiNbO_3_ Nanoparticles (LN NPs)

**Figure 1:**
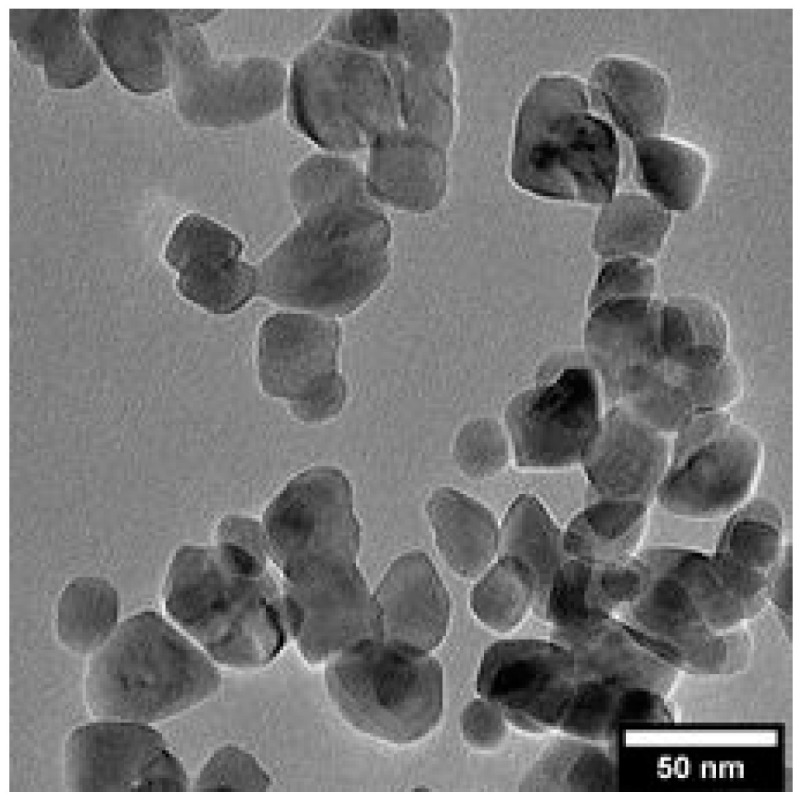
TEM images of LN NPs showing a pseudo-spherical shape.

The LN NPs were synthesized using a non-aqueous solvothermal method with 1,4-butanediol as co-solvent [32] and characterized following their crystalline phase, average diameter, size distribution, shape and degree of agglomeration. As described in Supplementary Materials (Figure S2), the X-ray diffractogram (XRD) indicated that the produced LN NPs are monocrystalline with the expected trigonal structure. From the full width at half maximum of the XRD peaks, a mean crystalline diameter of 45 nm was derived from the Scherrer formula, along the [110] crystallographic direction (Table S1, Supplementary Materials). The lowest dimension at 16 nm was found perpendicularly to the (006) diffraction peak thus giving an anistropic ratio of 2.6 [32]. The size and shape distribution of bare LN NPs was also obtained from TEM image analysis (Figure 1).

Using a sample size of 300 LN NPs, an average diameter of 34 ± 12 nm was determined. The pseudo-spherical shape was also noticed from the average degree of sphericity measured at 0.8. The difference between the Scherrer and TEM diameters arises from the random orientations of the LN NPs deposited on the TEM grid, their pseudo-spherical shape and average crystalline sizes derived from XRD data (Table S1, Supplementary Materials) along each [hkl] crystallographic direction [32]. As such, the diameter reported by XRD is larger than that obtained from TEM analysis. Additionally, the TEM diameter determination is limited by the relatively small sample size whereas the XRD analysis takes into account a higher number of NPs. The diameter of LN NPs derived from XRD data will be the one used in the following and the spherical morphology will be assumed to calculate an upper value of their volume. Note also that for comparison, the typical hydrodynamic diameter was measured at ~120 nm indicating the absence of agglomeration in the initial water-based aqueous suspensions. The corresponding hydrodynamic diameter of LN NPs is then 135 ± 15.7 nm. This latter value is significantly larger than the average diameters determined above as it considers the entire solvation sphere consisting of the ions in the Stern and diffuse layers around the LN NPs. In the following, the hydrodynamic diameter was monitored to account for the Au seeding and to qualitatively detect any possible agglomeration. In such a case, the measured hydrodynamic diameter we considered is above 300 nm. The zeta potential of water-based LN dispersions was determined to be –43.2 ± 3.6 mV, leading to a high stability of the LN NPs dispersed in aqueous media. This negative zeta potential value was attributed to the presence of residual OH surface groups [32].

### 3.2. Synthesis and Characterization of Au Seeds (AuSeeds)

Gold seeds (AuSeeds) were synthesized using a method that had been established since 1993 by Duff et al. [31] using tetrakis(hydroxymethyl)phosphonium chloride (THPC) as a reducer of Au^3+^ ions. In this method, the reaction to produce AuSeeds is done at pH 11.1. The presence of the OH^−^ ions is necessary for the neutralization reaction of the THPC to give tris(hydroxymethyl)phosphine (THP) and formaldehyde. Gold (III) chloride trihydrate, already dissolved in water, is hydrolyzed to give different AuCl*_x_*OH_4-*x*_ species [33]. The alkaline pH of the solution causes the Au(OH)_4_^−^ to be the predominant gold ion species. When the Au salt solution is added to the mixture of THP and formaldehyde, THP is able to partially reduce Au^3+^ to form THP-Au^+^ complexes, and the formaldehyde reduces these Au^+^ species to produce Au^0^ NPs [34]. Due to the complexation between THP and gold, phosphine groups could be expected at the surface of Au NPs, which would result in positively charged NPs. However, zeta potential measurements show a negative surface charge of –25.3 ± 8.0 mV, which is in agreement with the negative zeta potential values reported by Park et al. [35]. This negative charge is therefore thought to be due to the presence of surface AuCl_4_^−^ ions [36,37]. The geometric mean diameter of the spherical AuSeeds was determined to be 2.5 ± 0.5 nm by transmission electron microscopy (TEM, Figures S3a,b, Supplementary Materials). As expected, these ultra-small spherical Au NPs do not display a characteristic UV-visible absorption band (Figure S3c, Supplementary Materials). With the mean diameter and the spherical geometry, the volume of each seed was calculated to be 8.2 nm^3^. The mass of Au in one AuSeed is then 1.58 × 10^−19^ g using the density of Au (19.3 g/cm^3^). Assuming that all the Au^3+^ from the HAuCl_4_ is reduced to Au^0^ in the synthesis and consumed to form the AuSeeds, the total mass of produced Au^0^ was calculated to be 1.78 × 10^−3^ g. The corresponding theoretical concentration of AuSeeds was thus determined to be 0.037 g/L or 2.32 × 10^17^ NPs/L, which is in agreement with the order of magnitude of NPs concentration reported in the literature for this synthesis. In the review article by Garcıa-Soto *et al* [34] the particle concentration is reported to be between 10^17^ and 10^18^ particles per L. Consistently with the above calculations, our ICP-AES analysis resulted in a Au^0^ concentration of 0.028 g/L in the AuSeeds dispersion. This led to a number concentration of 1.77 × 10^17^ NPs/L when the average diameter determined by TEM is considered. The order of magnitude for the concentrations of AuSeeds determined by both methods is thus very consistent, the theoretical value only differing from the experimentally one by ~20%. In addition, because the ICP-AES analysis provides an accurate atomic quantification, the difference between the theoretical and experimental values indicated that all the Au^3+^ ions in the reaction are not reduced to Au^0^. The theoretical calculation thus only provides an acceptable estimation of the real AuSeeds concentration.

### 3.3. BPEI Polymer Surface Modification of LN NPs (LN@BPEI NPs)

Due to the negative surface charges of LN NPs (–43.2 ± 3.6 mV) and AuSeeds (–25.3 ± 8.0 mV), LN NPs had to undergo surface charge reversal in order to attach AuSeeds. In a study on the effect of the MW of BPEI polymer on the colloidal stability of spherical, 50 nm magnetite NPs, it was shown that among BPEI of MW 25,600 and 800 kDa, the 25 kDa BPEI modification allowed for the best colloidal dispersion and for the prevention of particle agglomeration [30]. In this work, the core diameter of LN NPs being 45 nm, the 25 kDa BPEI polymer was selected. Assuming that the loading capacity of LN@BPEI with AuSeeds would primarily depend on the number of surface protonated NH_2_, which can be altered by changing the pH of the solution, attention has been paid to the conditions of the AuSeed attachment. The degree of protonation of the NH_2_ moieties of BPEI polymer is approximately 44% at neutral pH (pH 7.5) [38] and increases at lower pH values. Here the BPEI attachment was carried out in neutral pH conditions leading to LN@BPEI NPs with an average zeta potential of 36.1 ± 2.9 mV. The average hydrodynamic diameter of LN@BPEI is then 159.6 ± 34.1 nm, i.e., the increase in the hydrodynamic diameter is 20% after BPEI addition. Also, the absence of free BPEI in the nanoparticle dispersion is essential to avoid the formation BPEI-AuSeed composite particles without the LN core. To determine the number of washing steps needed, an amine-based fluorescence analysis was thus performed according to the protocol adapted from the work of Khan et al. [39]. Fluorescamine, a non-fluorescent compound that upon reaction with primary amines, becomes fluorescent, was reacted with standard solutions of BPEI. Both BPEI and fluorescamine showed no fluorescence signal before mixing. Conversely, the fluorescamine-BPEI compound has a fluorescence maximum at 472 nm upon excitation at 388 nm. After plotting the intensity values at 472 nm for fluorescamine bound to BPEI, a standard concentration curve was then obtained (Figure S4, Supplementary Materials). The fluorescence spectrum of the supernatant of each washing step of the primary LN@BPEI dispersions showed a substantial decrease in the fluorescence intensity immediately after the first step, and by the third wash, a total suppression of the 472 nm band was observed (Figure S5, Supplementary Materials). The supernatant from the first washing had the highest content of fluorescamine-BPEI corresponding to a concentration of around 0.84 mg/mL. At lower concentrations of fluorescamine-BPEI, the emission spectra are less resolved so that by the third washing, the concentration of fluorescamine-BPEI in the supernatant was found constant at ~0.03 mg/mL for all the subsequent washing. This value was considered to be the lowest detection limit of the fluorescamine-BPEI species. This analysis demonstrated that there is still a significant amount of unbound BPEI present in the supernatant after the first washing step. Thus, the possibility of LN@BPEI NPs being present in the supernatant to then form LN@BPEI-fluorescamine NPs is not negligible but the risk was minimized by the successive centrifugations resulting in a pellet of NPs at the bottom of the centrifugation tube which was then re-dispersed by vigorous shaking and sonication. Using this fluorescence-based analysis, the concentration of BPEI present in the supernatants was determined and, consequently, the mass of BPEI that remained was assumed to be immobilized onto the NP surface. From this method, we estimated that approximately 1000–2000 BPEI polymer chains were attached to a single LN NP. XPS analysis also provided a quantification of BPEI molecules at the surface core of LN (Figure S6, Supplementary Materials). Nitrogen (N1s) was only observed in the LN@BPEI sample (Figure S6d, Supplementary Materials). The atomic ratio, Nb3d:N1s, was obtained at 5.26:3.09 (Table S2, Supplementary Materials). From this atomic ratio, the number of BPEI molecules per LN NP was estimated from its molecular structure (Figure S7, Supplementary Materials) at 1210, which corresponds to 23% wt of polymer to LN NP. In the work of Rosenholm et al. [40] a 18% wt of PEI polymer on SiO_2_ NPs was determined by thermogravimetry. The surface sensitivity of XPS analysis thus makes this technique well adapted to quantitatively assess the amount of adsorbed BPEI at the surface of LN NPs. Finally, both results, obtained from the fluorescamine-based titration and XPS methods, are in good agreement, further validating the fluorescence approach which is simpler and less expensive.

### 3.4. Attaching AuSeeds to BPEI-Modified LN NPs (LN@BPEI@AuSeeds NPs)

**Figure 2:**
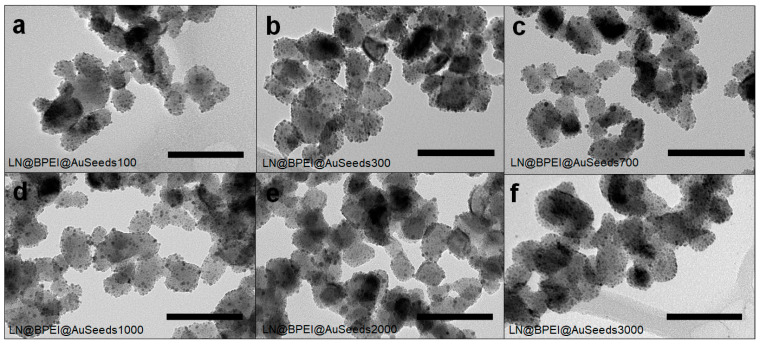
TEM images of gold seeds attached to LN NPs, at different gold seed loading densities. (**a**) LN@BPEI@AuScheme 100. (**b**) LN@BPEI@AuSeeds300, (**c**) LN@BPEI@AuSeeds700, (**d**) LN@BPEI@AuSeeds1000, (**e**) LN@BPEI@AuSeeds2000 and (**f**) LN@BPEI@AuSeeds3000. Scale bar corresponds to 100 nm.

The attachment of AuSeeds to the LN@BPEI NPs was then achieved by way of electrostatic and coordination interactions between the positive surface charge of the LN@BPEI NPs (+36.1 ± 2.9 mV) and the negative surface charge of the AuSeeds (–25.3 ± 8.0 mV). The controlled attachment of the AuSeeds to the LN is facilitated by the primary amine groups of BPEI. In addition to the electrostatic interactions between the protonated amines and the negative surface of AuSeeds, complexation reactions between the gold ion complexes located at the AuSeeds surface could also occur [41]. Considering that at neutral pH, BPEI is 44% charged leading to 213 NH_2_ moieties per polymer chain and that the XPS analysis gave an estimation of 1210 polymer chains per LN, the maximum number of available NH_2_ sites is 1.1 × 10^5^ for the AuSeeds attachment onto each LN NP. There are, however, additional considerations for the availability of these binding sites such as the surface conformation of BPEI molecules, steric hindrance and electrostatic repulsion between two negatively charged AuSeeds that will finally determine the minimum inter-seed distance. The maximum surface coverage achievable with the AuSeeds on the LN@BPEI surface was thus tested as follows: for a fixed number of LN@BPEI NPs and a varying concentration of AuSeeds in the dispersion, we assumed that the number of AuSeeds attached at the LN@BPEI surface would also vary. The following ratios of AuSeeds to LN@BPEI NP were thus prepared, 100:1, 300:1, 700:1, 1000:1, 2000:1 and 3000:1 (Table 1) and the corresponding samples labeled LN@BPEI@AuSeeds100, LN@BPEI@AuSeeds300, LN@BPEI@AuSeeds700, LN@BPEI@AuSeeds1000, LN@BPEI@AuSeeds2000 and LN@BPEI@AuSeeds3000 respectively. For all the tested AuSeeds concentrations, TEM imaging then revealed a random distribution of the AuSeeds at the surface of LN NP and different AuSeeds densities as shown in Figure 2.

**Figure 3:**
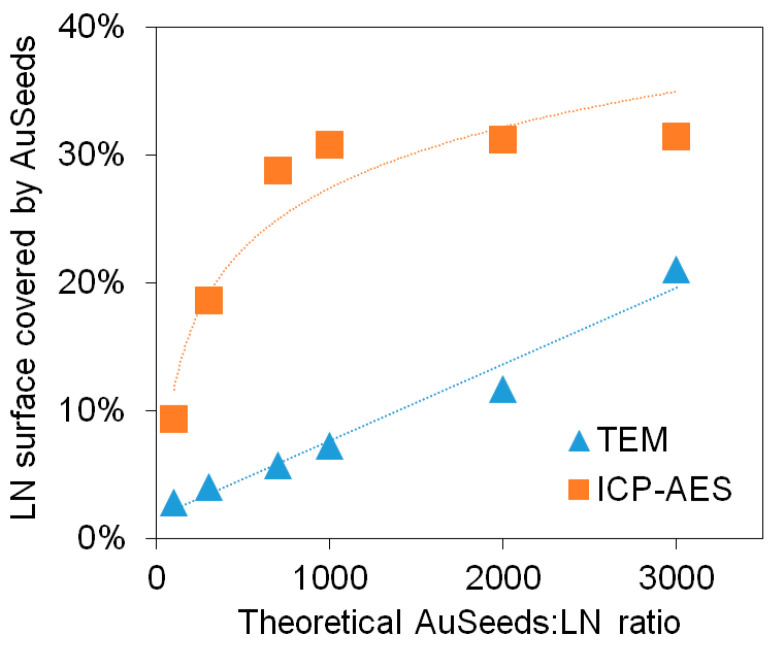
Percentage of the LN@BPEI surface covered by AuSeeds for each LN@BPEI@AuSeeds sample as determined by TEM imaging and ICP-AES analysis.

These results can be compared to those reported by De Silva Indrasekara et al. [25] where it was shown that the architecture of Au NPs attached to a SiO_2_ core NP was pH-dependent for Au NP diameters larger than 5 nm. In their work, at neutral pH, a random distribution of the Au NPs on the amine-terminated SiO_2_ core of 60 nm was observed for separately synthesized citrate-capped Au NPs of 5, 10 and 15 nm in diameter. This random distribution was obtained regardless of pH for the 5 nm Au NPs. Here, as Au NPs (AuSeeds) are 2.5 nm in diameter, the random distribution we observed is consistent with these previous observations. TEM imaging and ICP-AES analysis techniques were both employed to determine the percentage of the LN@BPEI surface covered by AuSeeds and this percentage was plotted as a function of the theoretical ratio of AuSeeds to LN (Figure 3). Details of calculations are provided in the Supplementary Materials.

**Figure 4:**
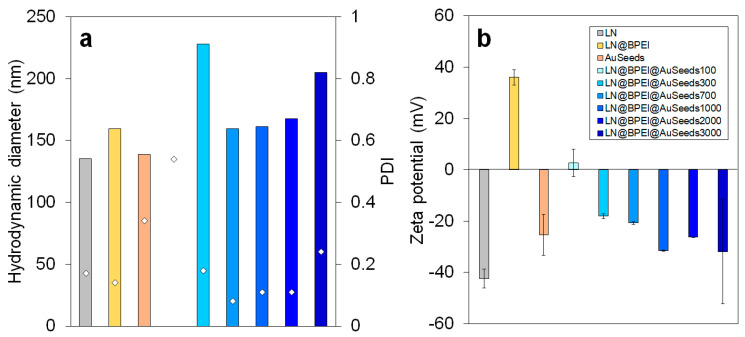
(**a**) Hydrodynamic diameters and PDI (◊ symbol, shown on the corresponding hydrodynamic diameter bars) values for LN, LN@BPEI, AuSeeds and LN@BPEI@AuSeeds NPs. The hydrodynamic diameter value obtained for sample LN@BPEI@AuSeeds100 was larger than 1000 nm due to a significant agglomeration and was thus omitted from the graph. (**b**) Zeta potential values for LN, LN@BPEI, AuSeeds and LN@BPEI@AuSeeds NPs.

From TEM analysis, the number of AuSeeds attached to each LN@BPEI core was found to increase linearly with the concentration of the AuSeeds dispersion and the as-obtained maximum surface coverage of LN@BPEI was approximately 21%, while results from ICP-AES revealed an asymptotic trend for theoretical AuSeeds:LN ratios above 1000 with LN@BPEI@AuSeeds3000 achieving the highest surface coverage at 31.5%. These different trends were expected as ICP-AES allows one to obtain a direct quantification of all the chemical elements present in the sample at a µg/L concentration range. It was thus considered more accurate than the TEM-based quantification, whose limitations are discussed in Supplementary Materials. The obtained surface coverage of 31.5% corresponds to 29% wt of Au. In the work of Goon et al. [30], 47.7% wt of Au on 50-nm cubic Fe_3_O_4_ NPs was determined by ICP-AES analysis. To better compare these results, the reported mass percentage was used to calculate the percent of Fe_3_O_4_ surface covered by Au NPs, and this percentage was determined to be 74%. An explanation for the difference in these achievable percentage surface coverages can be due to the shape of the NP cores. In the case of cubic-shaped Fe_3_O_4_ NPs, a larger surface area is to be considered comparatively to the pseudo-spherical LN NPs here studied whose diameter at 45 nm is similar to the side length of the cubic NPs. In addition, the cubic shape may also result in a different (possibly less compact) BPEI polymer configuration comparatively to the spherical one. This could lead to more NH_2_ terminal groups available for the AuSeeds attachment. The plateau observed in Figure 3 for the ICP-AES analysis thus indicates that the maximum surface coverage is already attained for the LN@BPEI@AuSeeds1000 sample. Raising the AuSeeds:LN ratio thus does not longer increase the coverage significantly. This was also noticed during the experimental process. After centrifugation of the LN@BPEI@AuSeeds100 to LN@BPEI@AuSeeds700 samples, a colorless supernatant was obtained whereas the supernatants from LN@BPEI@AuSeeds1000 to LN@BPEI@AuSeeds3000 series have, for the naked eye, the color of the initial AuSeeds dispersion as illustrated in Figure S3d (Supplementary Materials). Finally, we also emphasize that the overall accuracy on the surface coverage determination is also complicated with the inherent size and shape polydispersity of the primary LN cores as depicted in Figure 1 and Figure 2. Regarding DLS measurements, the hydrodynamic diameters and associated PDI values were found very acceptable though. As shown in Figure 4a, the hydrodynamic diameter is constant at about 150 nm for PDI values below 0.3 for all the LN and LN@BPEI@AuSeeds samples except for the LN@BPEI@AuSeeds100 sample which experienced a significant agglomeration and subsequent sedimentation.

The zeta potential values of LN, AuSeeds, LN@BPEI, and LN@BPEI@AuSeeds samples were also measured. The values reported in Figure 4b have been averaged after 30 measurements on each sample. Note that surface chemical modification of LN particles is well correlated with the zeta potential variation since the surface charge of bare LN NPs is first reversed after BPEI addition. Samples from LN@BPEI@AuSeeds100 to LN@BPEI@AuSeeds700 are prone to agglomeration and sedimentation in particular for the LN@BPEI@AuSeeds100 preparation whose zeta potential is very close to 0 mV. This weak surface charge is explained by the expected charge compensation between the positive BPEI and the negative AuSeeds. The agglomeration of NPs from LN@BPEI@AuSeeds100 to LN@BPEI@AuSeeds700 was also observed during the experimental process, NPs from those dispersions indeed settle down quickly at the bottom of their storage container after a few minutes. For the LN@BPEI@AuSeeds1000 to LN@BPEI@AuSeeds3000 series, samples remained well dispersed for one month as observed by the naked eye. Attachment of the AuSeeds at the LN@BPEI@AuSeeds surface was also found very stable under storage since individual AuSeeds could not be observed from TEM images 2 months after their preparation (Figure S8, Supplementary Materials).

### 3.5. Hyper Rayleigh Scattering of LN@BPEI@AuSeeds NPs

The microscopic entity used to quantitatively assess cross-section of the SHG process in nanoparticles is the optical first hyperpolarizability β that can be derived from hyper Rayleigh scattering (HRS) measurements. Intensity $I_{HRS}$ of NP dispersion can be expressed in the form of Equation (1):

(1) $$I_{HRS}=G\left(N_{s}\langle \beta_{s}^{2}\rangle +N_{np}\langle \beta_{np}^{2}\rangle \right)$$

where G contains all unnecessary constants, $N_{s}$ is the solvent concentration, $\beta_{s}$ is the solvent hyperpolarizability, here water, $N_{np}$ is the NP concentration and $\beta_{np}$ their first average hyperpolarizability after orientational averaging such that $\beta_{np}=\sqrt{\langle \beta_{np}^{2}\rangle }$. The internal reference method was used with $\beta_{s}=\sqrt{\langle \beta_{s}^{2}\rangle }=$ 0.087 × 10^−30^ esu after checking that pure water provides equal normalization [42].

**Figure 5:**
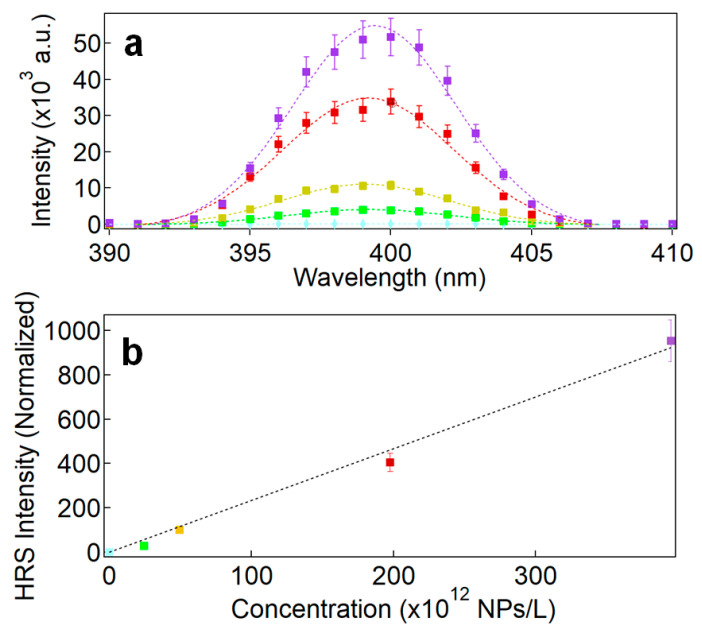
(**a**) Spectral response around 400 nm of the HRS intensities of LN NPs dispersions at different concentrations upon excitation with a vertically polarized wavelength at 800 nm. The corresponding NP concentrations are indicated in the lower panel whereas light blue dots stand for the spectral response of pure water. (**b**) Graphical plot of the normalized HRS intensities as a function of the NP concentration.

Following Equation (1), varying the NP concentration and measuring the corresponding HRS intensity normalized to that of the neat solvent yields the first hyperpolarizability from the slope of the line plot (Figure 5a,b). Note that correction for extinction, i.e., scattering and absorption, is performed using a linear extinction coefficient measurement at the fundamental and harmonic wavelength for each concentration. Also, the measured HRS intensities only display SHG signals without any contribution from other emission processes like fluorescence or photoluminescence. This was achieved with the recording of the HRS spectral band showing no broadband background light, as depicted in Figure 5a [43]. 

**Figure 6:**
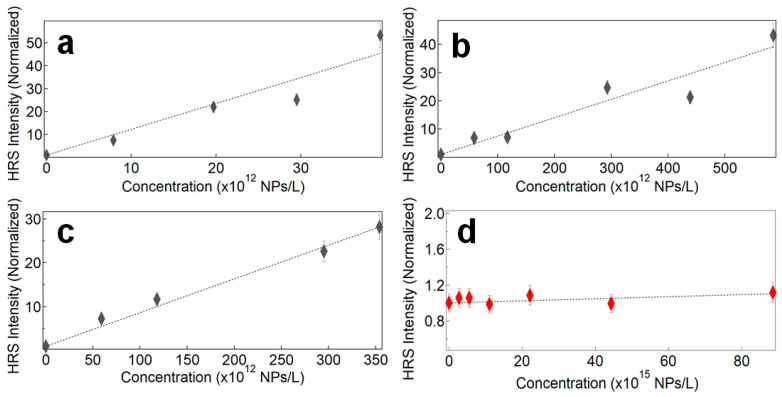
Normalized HRS intensities as a function of the NP concentration. (**a**) LN@BPEI@AuSeeds300, (**b**) LN@BPEI@AuSeeds1000, (**c**) LN@BPEI@AuSeeds3000, (**d**) AuSeeds.

The HRS intensities were also measured for the samples LN@BPEI@AuSeeds300, LN@BPEI@AuSeeds1000 and LN@BPEI@AuSeeds3000, as well as for the AuSeeds dispersions. Their normalized HRS intensities at different NP concentrations are plotted in Figure 6.

The corresponding β values for the AuSeeds and different LN@BPEI@AuSeeds NPs are all summarized in Table 2.

As the determination of $\beta_{np}$ requires precise knowledge of the NPs concentration, the initial concentration of LN was determined by weighing a dry powder amount and dispersing it in the necessary volume of water to adjust the desired mass concentrations, whereas the LN@BPEI@AuSeeds initial concentration was determined by ICP-AES analysis (refer to calculation in Supplementary Materials). The dispersions were then further diluted for the HRS measurements. The hyperpolarizability values describe the nonlinear optical response with a well-known volume scaling effect as for bare non-centrosymmetric oxide NPs, that influences the two-photon scattering cross-section [44,45]. After attachment of the AuSeeds to the LN@BPEI core NPs, the observed decrease in the hyperpolarizability $\beta_{np}$ values needs to be discussed according to the assumptions here made. Influence of the sample size polydispersity is first omitted since the mean NP volume of the LN core is considered, thereby neglecting the stronger contribution of larger diameter particles on the measured HRS intensities. Possible agglomeration effects are also not taken into account. Such effects would have been more pronounced for the sample LN@BPEI@AuSeeds300, whose zeta mean potential value is only of –18.0 mV. Besides, its measured hydrodynamic diameter above 200 nm (Figure 4a) indicates in this case a different agglomeration state comparatively to the other samples. Its hyperpolarizability value is however significantly larger than the ones obtained for LN@BPEI@AuSeeds1000 and LN@BPEI@AuSeeds3000. Taking into account that the surface coverage of AuSeeds for LN@BPEI@AuSeeds300 is around 18% and 31% for LN@BPEI@AuSeeds1000 and LN@BPEI@AuSeeds3000, respectively, the $\beta_{np}$ decrease seems to be correlated with a higher AuSeeds surface coverage. This unexpected, yet interesting, result raises the question of the AuSeeds impact on the SHG response of the LN core. It is suggested that a weak surface contribution to the SHG intensity remains, the volume origin dominating anyhow, for this size of the LN NPs. Interestingly, Table 2 results also suggest that a saturation appears beyond about a theoretical ratio of a thousand AuSeeds per LN@BPEI NP, which corresponds to a surface coverage percentage around 30%.

**Figure 7:**
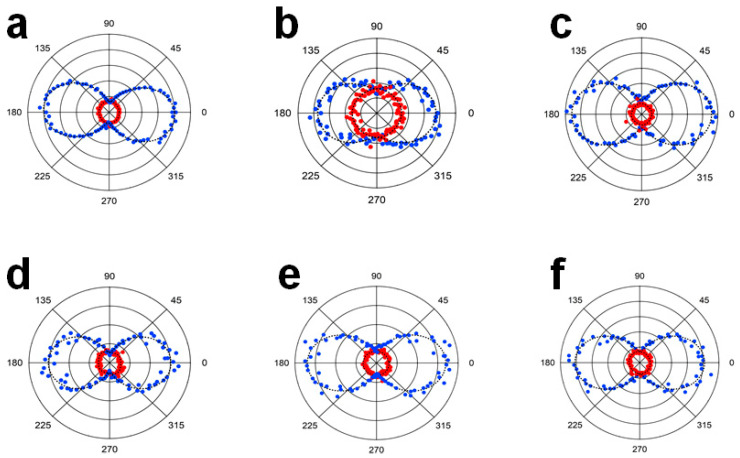
Polar plots of HRS intensities as a function of the polarization angles of the incident light. The blue and red data points correspond to vertical and horizontal harmonic polarization of the HRS intensities, respectively. (**a**) Core LN, (**b**) AuSeeds, (**c**) LN@BPEI@AuSeeds100, (**d**) LN@BPEI@AuSeeds300, (**e**) LN@BPEI@AuSeeds1000 and (**f**) LN@BPEI@AuSeeds3000.

To get further insights of the possible surface and volume contributions, polarization-resolved measurements were performed, as illustrated in Figure 7, after rotating the input polarization angle and collecting the vertically- and horizontally-polarized HRS intensities with respect to the horizontal plane of scattering (Figure S1, Supplementary Materials).

The depolarization ratio D was then calculated from Equation (2) for a vertical input polarization angle and from the HRS intensities $I_{HRS}^{H}\;$ and $I_{HRS}^{V}$ collected along the horizontal and vertical directions, respectively:

(2) $$D=\frac{I_{HRS}^{H}}{I_{HRS}^{V}}$$

The as-determined depolarization ratios are summarized in Table 3 for the different samples.

Interestingly, the measured depolarization ratio of the core LN sample is in perfect agreement with the calculated one of 0.13 from the non-zero elements of the second-order susceptibility tensor [15]. The first hyperpolarizability being a third-rank tensor, it can be decomposed into the sum of two irreducible tensors, a dipolar one noted $\beta^{\left(1\right)}$ and an octupolar one, $\beta^{\left(3\right)}$. A purely 1D single tensor element response confers to D the value of 0.2. Conversely, planar symmetric molecules of point group $D_{3h}$ have a depolarization factor of 2/3 [46]. Lithium niobate, which belongs to the point group $C_{3v}$, has a strong non-linearity along the c-axis and consequently, the nonlinear $d_{33}$ coefficient is largely dominant and well-above the other two non-zero elements (namely $d_{31}$ and $d_{22}$). The calculated and measured depolarization ratios are thus close to that of a pure single tensor element. Comparatively, the AuSeeds have a much larger D coefficient at 0.43 (Table 3) and the reason for this is the surface origin of their response as gold is a centrosymmetric material. Their response is therefore highly three-dimensional and strongly dependent on their geometry [47]. For the LN@BPEI@AuSeeds samples, despite their very similar response to the bare LN NPs (Table 3), a weak change in the depolarization ratio is observed, as a result of the weak surface contribution due to the AuSeeds. We can also notice that the higher agglomeration state of LN@BPEI@AuSeeds100 compared to the other LN@BPEI@AuSeeds samples does not seem to have any influence on the depolarization ratio. The random distribution of the Au Seeds at the LN NPS surface, as seen from TEM images of Figure 2, should lead to an increase of the depolarization ratio, that is a reinforcement of the octupolar response as compared to the dipolar one of the bare LN NPs. This effect is however small due to the weakness of the AuSeeds hyperpolarizability. An inhomogeneous distribution of the AuSeeds could in principle alter this situation though [48]. The stronger HRS response of LN thus dominates the measured HRS intensities for the hybrid LN@BPEI@AuSeeds NPs. Therefore it is still yet to be understood why the addition of AuSeeds to the surface of LN decreased the β values.

## 4. Conclusions

Starting from pseudo-spherical LN nanoparticles of 45 nm in diameter, a BPEI polymer of 25,000 g/mol was first selected to induce a surface charge reversal and, because of the available surface NH_2_ groups, for the subsequent attachment of negatively-charged AuSeeds by means of both electrostatic and coordination interactions. According to the AuSeeds:LN ratio, the percentage of the LN@BPEI surface that can be covered by the AuSeeds can be changed, with a maximum surface coverage of 31% as determined by ICP-AES analysis. At low amounts of AuSeeds corresponding to surface coverage below 19%, charge compensation between the BPEI and AuSeeds results in weakly charged LN@BPEI@AuSeeds prone to agglomeration and sedimentation processes. Conversely, at the maximum surface coverage of 31%, colloidal dispersions have been obtained. The quadratic nonlinear optical properties of the seeded and unseeded nanoparticles were then determined from HRS experiments. It is observed that the first hyperpolarizability revealed the decrease of SHG response of the LN NPs decrease upon the attachment of AuSeeds to its surface. This result indicates that this surface modification lends to the LN@BPEI NPs weak surface contribution that is detected. Therefore, the SHG response may only increase if a complete plasmonic shell is built, as reported in the literature, or, possibly, if an inhomogeneous distribution of the AuSeeds is realized.

## Figures and Tables

**Table 1:** Volumes and final concentrations of the AuSeeds dispersions used for varying the density of AuSeeds attached to the LN@BPEI surface. The stock AuSeeds dispersion had a concentration of 2.32 × 10^17^ NPs/L. For each sample, 1 mL of 1.58 × 10^15^ NPs/L of LN@BPEI was used.

Sample Name	Volume of AuSeeds Dispersion (mL)	Volume of H_2_O (mL)	Final AuSeeds Concentration (NPs/L)	Theoretical AuSeeds:LN Ratio
LN@BPEI@AuSeeds100	0.349	10.239	6.98 × 10^15^	100
LN@BPEI@AuSeeds300	1.046	9.542	2.09 × 10^16^	300
LN@BPEI@AuSeeds700	2.441	8.147	4.88 × 10^16^	700
LN@BPEI@AuSeeds1000	3.486	7.102	6.97 × 10^16^	1000
LN@BPEI@AuSeeds2000	6.973	3.615	1.39 × 10^17^	2000
LN@BPEI@AuSeeds3000	10.459	0.129	2.09 × 10^17^	3000

**Table 2:** First hyperpolarizability values determined from the slope of the HRS intensity plots vs the NP concentration for AuSeeds, LN@BPEI@AuSeeds300, LN@BPEI@AuSeeds1000, LN@BPEI@AuSeeds3000 and the bare LN NPs of 45 nm.

Sample Name	$\sqrt{\langle \beta_{np}^{2}\rangle }\;(\mathrm{esu})$
AuSeeds	(5.47 ± 3.4) × 10^−28^
LN@BPEI@AuSeeds300	(0.53 ± 0.2) × 10^−24^
LN@BPEI@AuSeeds1000	(0.13 ± 0.04) × 10^−24^
LN@BPEI@AuSeeds3000	(0.14 ± 0.03) × 10^−24^
LN	(0.77 ± 0.14) × 10^−24^

**Table 3:** Depolarization ratios for the core LN, AuSeeds and different LN@BPEI@AuSeeds samples.

Sample Name	D
LN	0.16 ± 0.01
LN@BPEI@AuSeeds100	0.2 ± 0.02
LN@BPEI@AuSeeds300	0.17 ± 0.02
LN@BPEI@AuSeeds1000	0.18 ± 0.03
LN@BPEI@AuSeeds3000	0.18 ± 0.03
AuSeeds	0.43 ± 0.1

## Supplementary Materials

The following are available online at https://www.mdpi.com/article/10.3390/nano11040950/s1, Figure S1: Illustration of the experimental set-up for the Hyper Rayleigh Scattering measurements and the determination of the first hyperpolarizability of LN@BPEI@AuSeeds NPs, Figure S2: Comparison between the X-ray diffractogram of LN NPs (black) and the LiNbO_3_ reference ICSD pattern #80628 for LiNbO_3_ bulk crystal of stoichiometric composition, Figure S3: (a) Representative TEM image of the synthesized spherical AuSeeds, (b) histogram showing the diameter distribution of the AuSeeds, (c) UV-visible absorption spectrum of AuSeeds and (d) photograph of AuSeeds dispersion, Figure S4: (a) Fluorescence spectra of fluorescamine bound to BPEI at different standard BPEI concentrations for an excitation wavelength at 388 nm and (b) corresponding calibration curve obtained from the fluorescence intensity at 472 nm, Figure S5: Fluorescence spectra of the supernatants after each washing step of the BPEI coated LN NPs. Excitation wavelength set at 388 nm, Figure S6: XPS survey spectra of (a) LN and (b) LN@BPEI NPs. N1s core level (shown by a red square in Figures S6a and S6b) of (c) LN and (d) LN@BPEI NPs, Figure S7: Molecular formula of Branched PolyEthylenImine (BPEI). Image retrieved from Sigma Aldrich BPEI product information website, Figure S8: Comparison of TEM images from (a) freshly-prepared LN@BPEI@AuSeeds NPs and (b) after a 2-month storage. Scale bar corresponds to 50 nm, Calculation of the number of BPEI molecules per LN NP from XPS results, Calculation of average number of AuSeeds per LN NP from ICP-AES results, Calculation of the percentage of LN surface covered by AuSeeds, Discussion on the limitations of TEM-based quantification compared to ICP-AES analysis, Expression of the theoretical depolarization ratio D for LiNbO_3_, Table S1: Scherrer diameters measured from XRD along the different crystallographic directions, Table S2: XPS binding energies and atomic percentages for the LN@BPEI NPs.

## References

[1] Lattuada M., Hatton T.A. (2011). Synthesis, properties and applications of Janus nanoparticles. Nano Today.

[2] Chatterjee K., Sarkar S., Rao K.J., Paria S. (2014). Core/shell nanoparticles in biomedical applications. Adv. Colloid Interface Sci..

[3] Purbia R., Paria S. (2015). Yolk/shell nanoparticles: Classifications, synthesis, properties, and applications. Nanoscale.

[4] Walker J.M., Zaleski J.M. (2016). A simple route to diverse noble metal-decorated iron oxide nanoparticles for catalysis. Nanoscale.

[5] Asif M., Aziz A., Ashraf G., Wang Z., Wang J., Azeem M., Chen X., Xiao F., Liu H. (2018). Facet-Inspired Core–Shell Gold Nanoislands on Metal Oxide Octadecahedral Heterostructures: High Sensing Performance toward Sulfide in Biotic Fluids. ACS Appl. Mater. Interfaces.

[6] Lee D.K., Song Y., Tran V.T., Kim J., Park E.Y., Lee J. (2017). Preparation of concave magnetoplasmonic core-shell supraparticles of gold-coated iron oxide via ion-reducible layer-by-layer method for surface enhanced Raman scattering. J. Colloid Interface Sci..

[7] Westcott S.L., Oldenburg S.J., Lee A.T.R., Halas N.J. (1998). Formation and Adsorption of Clusters of Gold Nanoparticles onto Functionalized Silica Nanoparticle Surfaces. Langmuir.

[8] JoŢca J., Harmel J., Joanny L., Ryzhikov A., Kahn M.L., Fau P., Chaudret B., Fajerwerg K., Jońca J. (2017). Au/MOx (M = Zn, Ti) nanocomposites as highly efficient catalytic filters for chemical gas sensing at room temperature and in humid atmosphere. Sens. Actuators B Chem..

[9] Qian Z., Hastings S.P., Li C., Edward B., McGinn C.K., Engheta N., Fakhraai Z., Park S.-J. (2015). Raspberry-like Metamolecules Exhibiting Strong Magnetic Resonances. ACS Nano.

[10] Randrianalisoa J., Li X., Serre M., Qin Z. (2017). Understanding the Collective Optical Properties of Complex Plasmonic Vesicles. Adv. Opt. Mater..

[11] Sánchez-Dena O., Villalobos-Mendoza S.D., Farías R., Fierro-Ruiz C.D. (2020). Lithium Niobate Single Crystals and Powders Reviewed—Part II. Crystals.

[12] Staedler D., Magouroux T., Rachid H., Joulaud C., Extermann J., Schwung S., Passemard S., Kasparian C., Clarke G., Gerrmann M. (2012). Harmonic Nanocrystals for Biolabeling: A Survey of Optical Properties and Biocompatibility. ACS Nano.

[13] Vuilleumier J., Gaulier G., De Matos R., Mugnier Y., Campargue G., Wolf J.-P., Bonacina L., Gerber-Lemaire S. (2019). Photocontrolled Release of the Anticancer Drug Chlorambucil with Caged Harmonic Nanoparticles. Helvetica Chim. Acta.

[14] Campargue G., La Volpe L., Giardina G., Gaulier G., Lucarini F., Gautschi I., Le Dantec R., Staedler D., Diviani D., Mugnier Y. (2020). Multiorder Nonlinear Mixing in Metal Oxide Nanoparticles. Nano Lett..

[15] Riporto J., Urbain M., Mugnier Y., Multian V., Riporto F., Bredillet K., Beauquis S., Galez C., Monnier V., Chevolot Y. (2019). Second harmonic spectroscopy of ZnO, BiFeO_3_ and LiNbO_3_ nanocrystals. Opt. Mater. Express.

[16] Hentschel M., Metzger B., Knabe B., Buse K., Giessen H. (2016). Linear and nonlinear optical properties of hybrid metallic–dielectric plasmonic nanoantennas. Beilstein J. Nanotechnol..

[17] Linnenbank H., Grynko Y., Förstner J., Linden S. (2016). Second harmonic generation spectroscopy on hybrid plasmonic/dielectric nanoantennas. Light. Sci. Appl..

[18] Richter J., Steinbrück A., Pertsch T., Tünnermann A., Grange R. (2012). Plasmonic Core–Shell Nanowires for Enhanced Second-Harmonic Generation. Plasmonics.

[19] Mattoli V., Farrokhtakin E., Ciofani G., Puleo G.L., De Vito G., Filippeschi C., Mazzolai B., Piazza V. (2013). Barium titanate core – gold shell nanoparticles for hyperthermia treatments. Int. J. Nanomed..

[20] Richter J., Steinbrück A., Zilk M., Sergeyev A., Pertsch T., Tünnermann A., Grange R. (2014). Core–shell potassium niobate nanowires for enhanced nonlinear optical effects. Nanoscale.

[21] Zhang Y., Manjavacas A., Hogan N.J., Zhou L., Orozco C.A., Dong L., Day J.K., Nordlander P., Halas N.J. (2016). Toward Surface Plasmon-Enhanced Optical Parametric Amplification (SPOPA) with Engineered Nanoparticles: A Nanoscale Tunable Infrared Source. Nano Lett..

[22] Wang Y., Barhoumi A., Tong R., Wang W., Ji T., Deng X., Li L., Lyon S.A., Reznor G., Zurakowski D. (2018). BaTiO_3_-core Au-shell nanoparticles for photothermal therapy and bimodal imaging. Acta Biomater..

[23] Pu Y., Grange R., Hsieh C.-L., Psaltis D. (2010). Nonlinear Optical Properties of Core-Shell Nanocavities for Enhanced Second-Harmonic Generation. Phys. Rev. Lett..

[24] Galletto P., Brevet P.F., Girault H.H., Antoine R., Broyer M. (1999). Enhancement of the Second Harmonic Response by Adsorbates on Gold Colloids: The Effect of Aggregation. J. Phys. Chem. B.

[25] Indrasekara A.S.D.S., Norton S.J., Geitner N.K., Crawford B.M., Wiesner M.R., Vo-Dinh T. (2018). Tailoring the Core–Satellite Nanoassembly Architectures by Tuning Internanoparticle Electrostatic Interactions. Langmuir.

[26] Bhana S., Rai B.K., Mishra S.R., Wang Y., Huang X. (2012). Synthesis and properties of near infrared-absorbing magnetic–optical nanopins. Nanoscale.

[27] Neuberg P., Kichler A. (2014). Recent Developments in Nucleic Acid Delivery with Polyethylenimines. Nonviral Vectors for Gene Therapy- Lipid- and Polymer-Based Gene Transfer.

[28] Nimesh S. (2013). Chapter 10: Polyethylenimine nanoparticles. Gene Therapy—Potential Applications of Nanotechnology.

[29] Lu W., Ling M., Jia M., Huang P., Li C., Yan B. (2014). Facile synthesis and characterization of polyethylenimine-coated Fe_3_O_4_ superparamagnetic nanoparticles for cancer cell separation. Mol. Med. Rep..

[30] Goon I.Y., Lai L.M.H., Lim M., Munroe P., Gooding J.J., Amal R. (2009). Fabrication and Dispersion of Gold-Shell-Protected Magnetite Nanoparticles: Systematic Control Using Polyethyleneimine. Chem. Mater..

[31] Duff D.G., Baiker A., Edwards P.P. (1993). A new hydrosol of gold clusters. 1. Formation and particle size variation. Langmuir.

[32] Urbain M., Riporto F., Beauquis S., Monnier V., Marty J.-C., Galez C., Durand C., Chevolot Y., Le Dantec R., Mugnier Y. (2021). On the Reaction Pathways and Growth Mechanisms of LiNbO_3_ Nanocrystals from the Non-Aqueous Solvothermal Alkoxide Route. Nanomaterials.

[33] Wang S., Qian K., Bi X., Huang W. (2009). Influence of Speciation of Aqueous HAuCl_4_ on the Synthesis, Structure, and Property of Au Colloids. J. Phys. Chem. C.

[34] García-Soto M.J., González-Ortega O. (2016). Synthesis of silica-core gold nanoshells and some modifications/variations. Gold Bull..

[35] Park S., Park M., Han P., Lee S. (2007). Relative Contributions of Experimental Parameters to NIR-Absorption Spectra of Gold Nanoshells. J. Ind. Eng. Chem..

[36] Li Q., Zheng A.J., Liu Z. (2003). Site-Selective Assemblies of Gold Nanoparticles on an AFM Tip-Defined Silicon Template. Langmuir.

[37] Pei L., Mori K., Adachi M. (2004). Formation Process of Two-Dimensional Networked Gold Nanowires by Citrate Reduction of AuCl4-and the Shape Stabilization. Langmuir.

[38] Curtis K.A., Miller D., Millard P., Basu S., Horkay F., Chandran P.L. (2016). Unusual Salt and pH Induced Changes in Polyethylenimine Solutions. PLoS ONE.

[39] Alam Khan F., Akhtar S., Almofty S.A., Almohazey D., AlOmari M. (2018). FMSP-Nanoparticles Induced Cell Death on Human Breast Adenocarcinoma Cell Line (MCF-7 Cells): Morphometric Analysis. Biomolecules.

[40] Rosenholm J.M., Meinander A., Peuhu E., Niemi R., Eriksson J.E., Sahlgren C.C., Lindén M. (2008). Targeting of Porous Hybrid Silica Nanoparticles to Cancer Cells. ACS Nano.

[41] Kumar A., Mandal S., Selvakannan P.R., Pasricha R., Mandale A.B., Sastry M. (2003). Investigation into the Interaction between Surface-Bound Alkylamines and Gold Nanoparticles. Langmuir.

[42] Duboisset J., Matar G., Russier-Antoine I., Benichou E., Bachelier G., Jonin C., Ficheux D., Besson F., Brevet P.F. (2010). First Hyperpolarizability of the Natural Aromatic Amino Acids Tryptophan, Tyrosine, and Phenylalanine and the Tripeptide Lysine−Tryptophan−Lysine Determined by Hyper-Rayleigh Scattering. J. Phys. Chem. B.

[43] De Meulenaere E., De Coene Y., Russier-Antoine I., Vanpraet L., Haute C.V.D., Thevissen K., Baekelandt V., Bartic C., Hofkens J., Brevet P.-F. (2020). Fluorescence-free First Hyperpolarizability Values of Fluorescent Proteins and Channel Rhodopsins. J. Photochem. Photobiol. A Chem..

[44] Le Dantec R., Mugnier Y., Djanta G., Bonacina L., Extermann J., Badie L., Joulaud C., Gerrmann M., Rytz D., Wolf J.P. (2011). Ensemble and Individual Characterization of the Nonlinear Optical Properties of ZnO and BaTiO_3_ Nanocrystals. J. Phys. Chem. C.

[45] Kim E., Steinbrück A., Buscaglia M.T., Buscaglia V., Pertsch T., Grange R. (2013). Second-Harmonic Generation of Single BaTiO_3_ Nanoparticles down to 22 nm Diameter. ACS Nano.

[46] Boyd R.W. (2008). Nonlinear Optics.

[47] Duboisset J., Brevet P.-F. (2019). Second-Harmonic Scattering-Defined Topological Classes for Nano-Objects. J. Phys. Chem. C.

[48] Hayakawa T., Usui Y., Bharathi S., Nogami M. (2004). Second Harmonic Generation from Coupled Surface-Plasmon Resonances in Self-Assembled Gold-Nanoparticle Monolayers Coated with an Aminosilane. Adv. Mater..

