# Supplementary material for: Gold-seeded Lithium Niobate Nanoparticles: Influence of Gold Surface Coverage on Second Harmonic Properties
Source: Nanomaterials (Basel). 2021 Apr 8;11(4):950. doi: 10.3390/nano11040950 (PMC8068263; doi:10.3390/nano11040950)
Supplement: Supplementary file 1 [file nanomaterials-11-00950-s001.pdf]

Supplementary Materials

Gold-seeded lithium niobate nanoparticles: influence of gold surface coverage on second harmonic properties

Rachael Taitt[1], Mathias Urbain[2], Zacharie Behel[3], Ana-María Pablo-Sainz-Ezquerra[1], Iryna Kandybka[1], Eloïse Millet[1], Nicolas Martinez-Rodriguez[1], Christelle Yeromonahos[1], Sandrine Beauquis[2], Ronan Le Dantec[2], Yannick Mugnier[2], Pierre-François Brevet[3], Yann Chevolot[1], Virginie Monnier[1]*

[1] Univ Lyon, ECL, INSA Lyon, CNRS, UCBL, CPE Lyon, INL, UMR5270, 69130 Ecully, France Université de Lyon, Ecole Centrale de Lyon, UMR CNRS 5270, Institut des Nanotechnologies de Lyon, Ecully, F-69130, France.

[2] Université Savoie Mont Blanc, SYMME, F-74000 Annecy, France.

[3] Université de Lyon, Université Claude Bernard Lyon 1, UMR CNRS 5306, Institut Lumière Matière, F-69622, Villeurbanne, France.

*E-mail: virginie.monnier@ec-lyon.fr

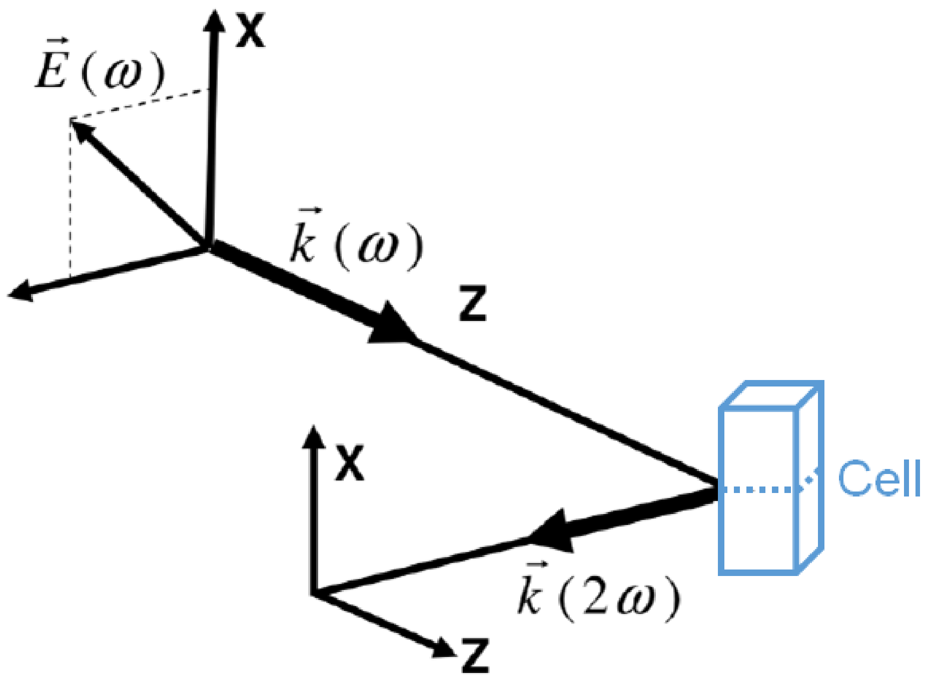

Figure S1. Illustration of the experimental set-up for the Hyper Rayleigh Scattering measurements and the determination of the first hyperpolarizability of LN@BPEI@AuSeeds NPs.

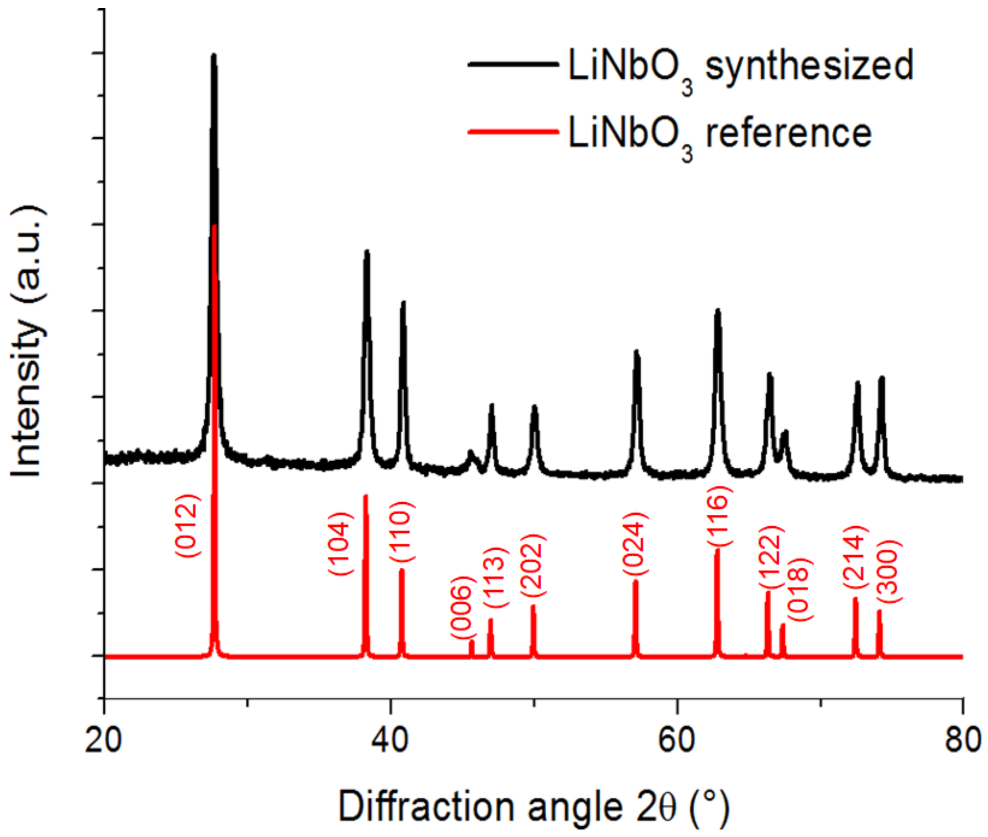

Figure S2. Comparison between the X-ray diffractogram of LN NPs (black) and the $LiNbO_3$ reference ICSD pattern #80628 for $LiNbO_3$ bulk crystal of stoichiometric composition.[1]

Crystallographic direction	Scherrer diameter (nm)
[012]	27
[104]	26
[110]	45
[006]	12
[113]	32
[202]	31
[024]	29
[116]	29

Table S1. Scherrer diameters measured from XRD along the different crystallographic directions.

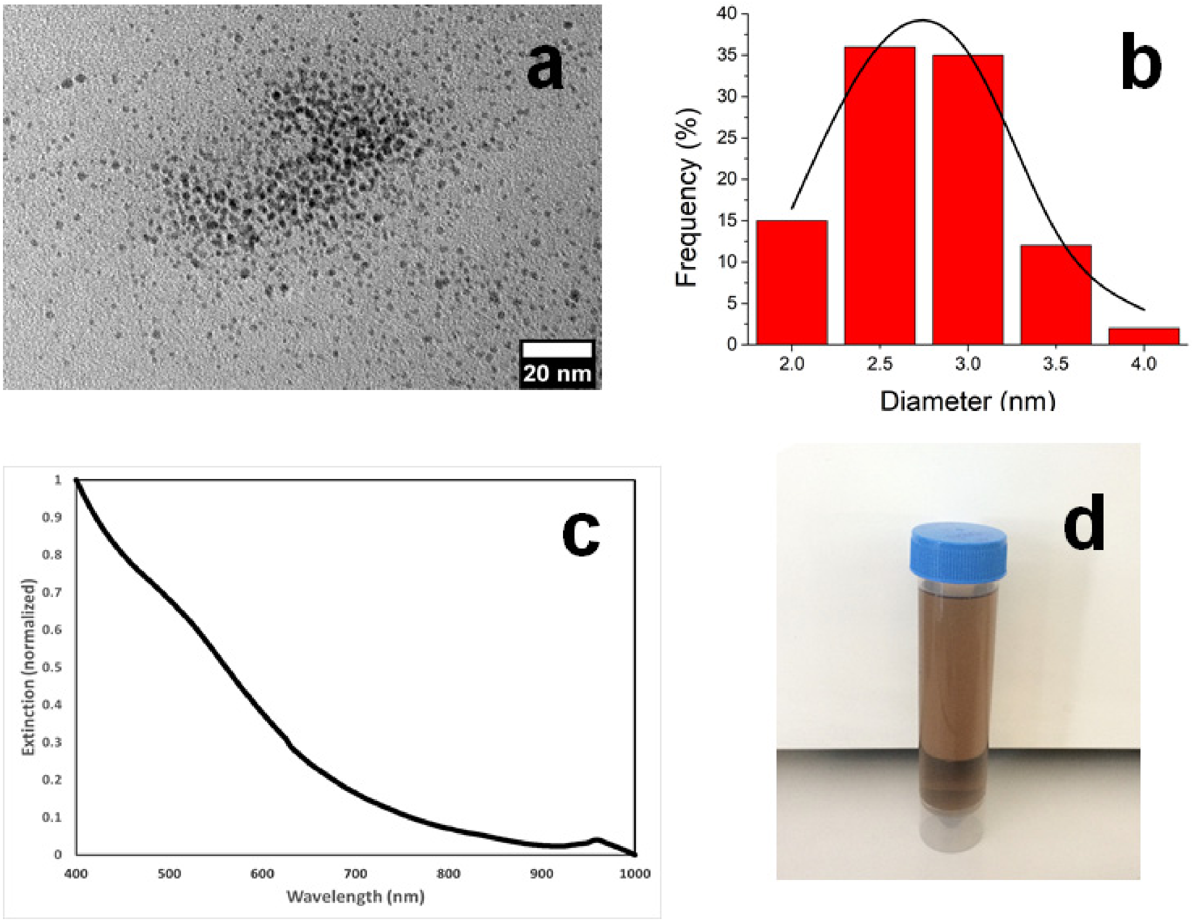

Figure S3. (a) Representative TEM image of the synthesized spherical AuSeeds, (b) histogram showing the diameter distribution of the AuSeeds, (c) UV-visible absorption spectrum of AuSeeds and (d) photograph of AuSeeds dispersion.

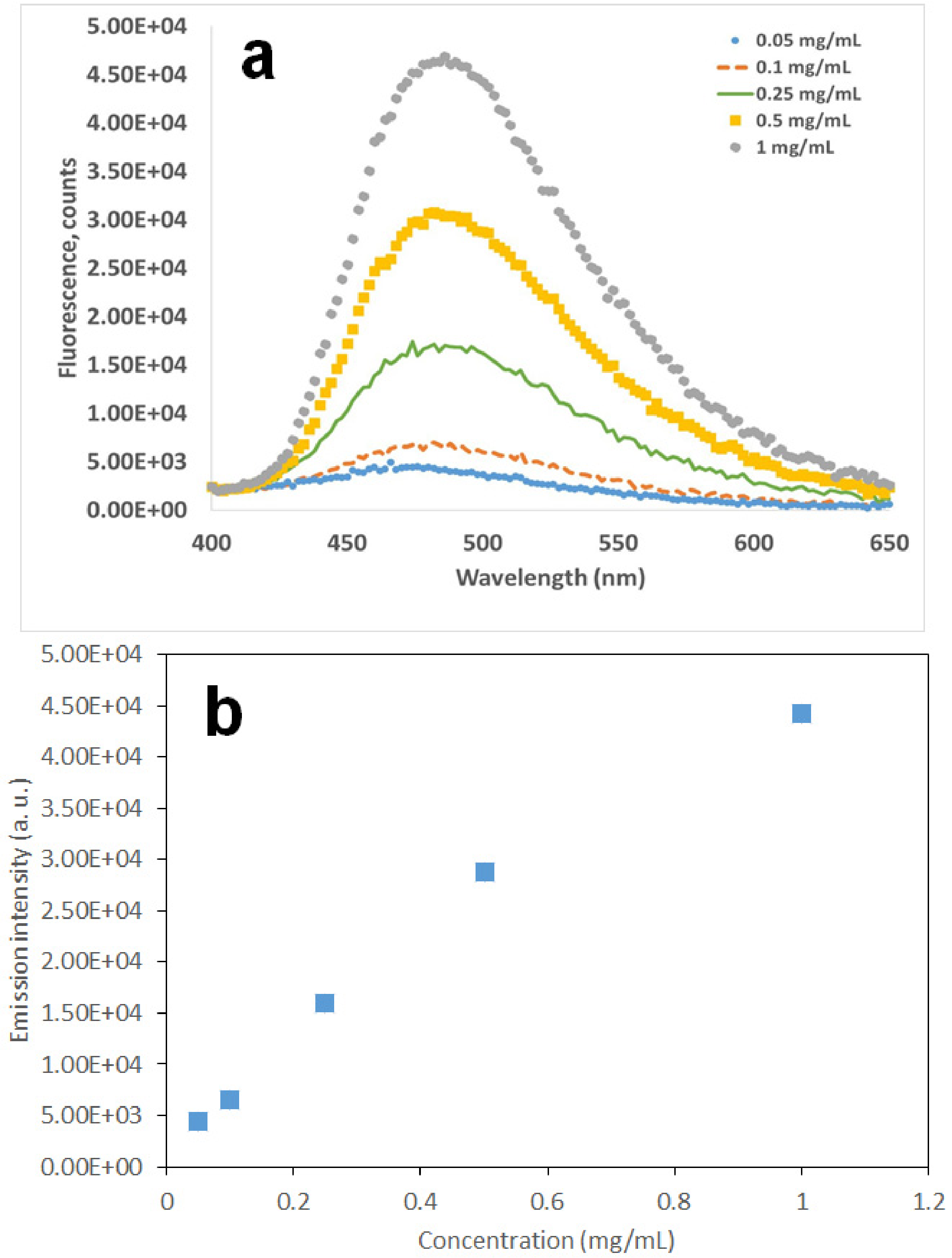

Figure S4. (a) Fluorescence spectra of fluorescamine bound to BPEI at different standard BPEI concentrations for an excitation wavelength at 388 nm and (b) corresponding calibration curve obtained from the fluorescence intensity at 472 nm.

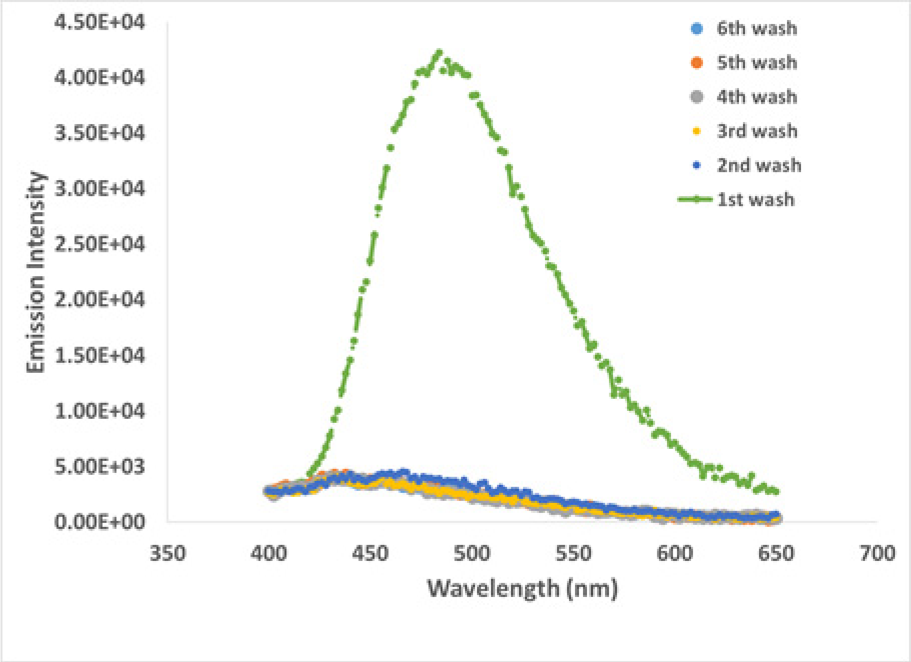

Figure S5. Fluorescence spectra of the supernatants after each washing step of the BPEI coated LN NPs. Excitation wavelength set at 388 nm.

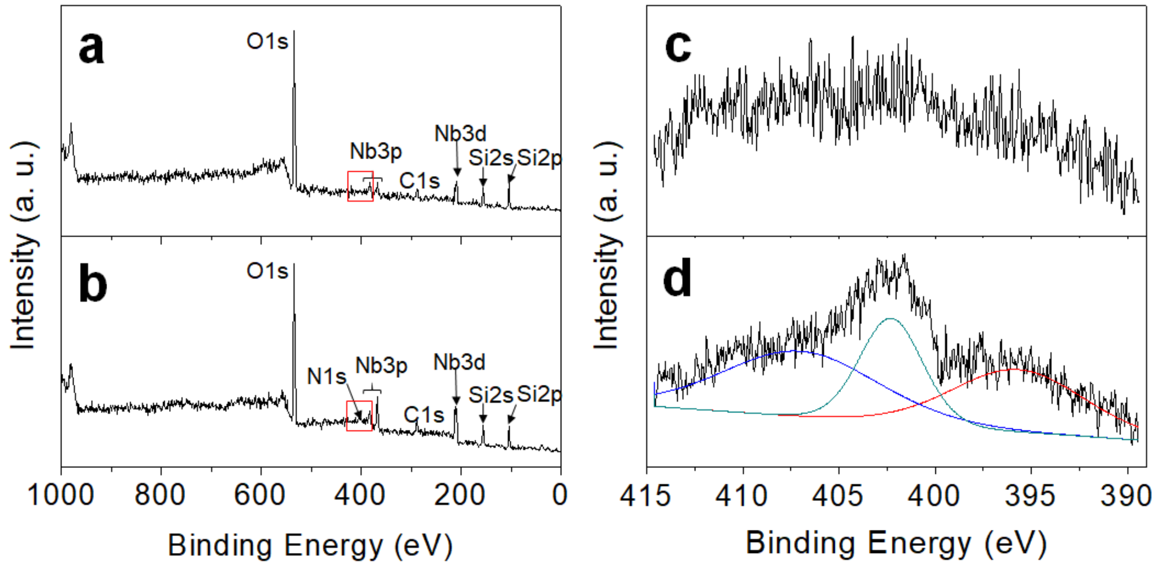

Figure S6. XPS survey spectra of (a) LN and (b) LN@BPEI NPs. N1s core level (shown by a red square in Figures S6a and S6b) of (c) LN and (d) LN@BPEI NPs.

Peak name	Binding energy (eV)	Atomic percentage (%)
Nb 3d	210.346	5.26
O 1s	533.332	47.64
N 1s	402.713	3.09
C 1s	288.717	15.42
Si 2p	104.663	23.85
Li 1s	64.2894	4.74

Table S2. XPS binding energies and atomic percentages for the LN@BPEI NPs.

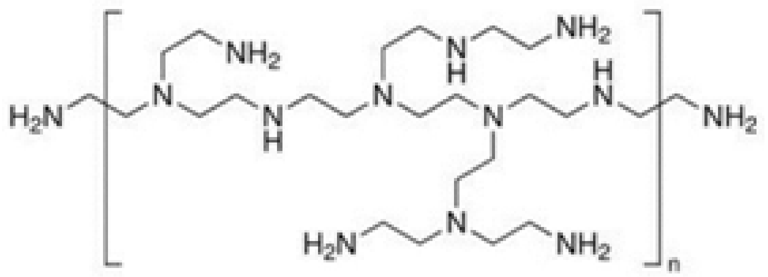

Figure S7. Molecular structure of Branched PolyEthylenImine (BPEI). Image retrieved from Sigma Aldrich BPEI product information website.[2]

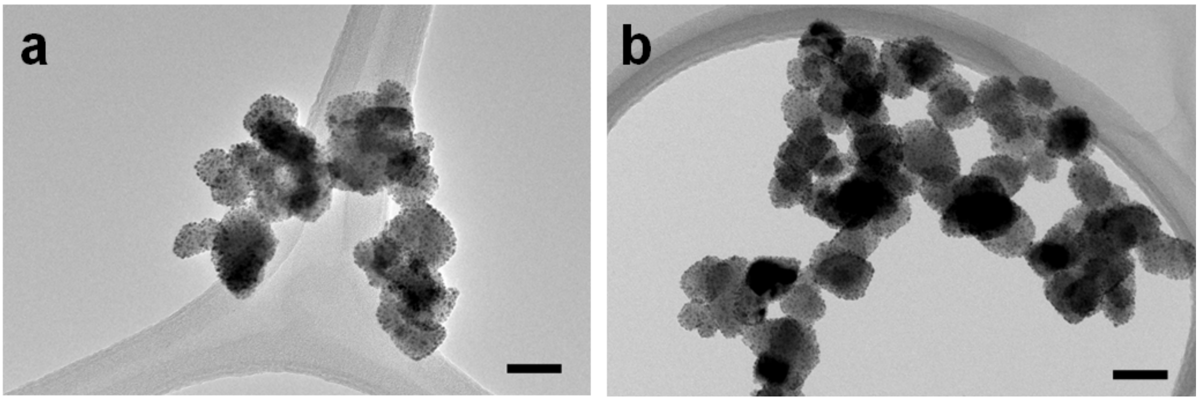

Figure S8. Comparison of TEM images from (a) freshly-prepared LN@BPEI@AuSeeds NPs and (b) after a 2-month storage. Scale bar corresponds to 50 nm.

Calculation of the number of BPEI molecules per LN NP from XPS results.

The maximum analysis depth in XPS was taken to be 10 nm and a spherical geometry was assumed for LN NPs of diameter 45 nm. The surface area of a single NP was thus calculated to be 6362 nm^2 from which the volume of each LN NP analyzed by XPS was determined to be 6.36×10^{-4} nm^3 considering that the probed volume per LN particle is the product of the XPS analysis depth by the surface area of a single LN NP. Then, using the density of $LiNbO_3$ (4.65 g/cm^3), the mass of $LiNbO_3$ per particle that was analyzed by XPS was calculated to be 2.96×10^{-16} g. From the mass percent of Li, Nb and O in $LiNbO_3$ compound at 5%, 63% and 32%, respectively, and the calculated mass of $LiNbO_3$, the mass of Nb atoms per LN particle thus probed was calculated to be 1.85×10^{-16} g, namely 1.2×10^6 Nb atoms. The molecular structure of BPEI is shown in Figure S7. The molecular weight of a polymer is the summation of all the molar masses of the atoms present in one polymer chain. Therefore, starting from the MW of the 25 kDa BPEI, the number of monomer units M that comprises of a single chain was calculated from Eq. S1.

$$M = \frac{25000 - (2 \times MW(NH_2) + 2 \times MW(CH_2))}{MW(1\ repeat\ unit)} = \frac{25000 - 60}{473} = 53 \quad \text{(Eq. S1)}$$

where MW is the molecular weight.

From the number of monomer units per polymer chain, the number of N atoms per polymer chain could then be calculated from Eq. S2.

$$N = (M \times 11) + 2 = 585 \quad \text{(Eq. S2)}$$

Returning to the stoichiometric ratio of Nb to N as determined by XPS (5.26:3.09), and having calculated the number of Nb atoms per LN analyzed by XPS (1.2×10^6 atoms), the number of N atoms per LN NP was calculated to be 7.08×10^5. As the only source of N atoms is from BPEI, the number of polymer chain molecules present at the LN NP surface can be calculated by dividing this value by the number of N atoms in one polymer chain. From this calculation we obtain 1210 polymer chains per LN NP, which corresponds to 23% wt of polymer to LN NP.

Calculation of average number of AuSeeds per LN NP from TEM images.

For the quantification based on TEM imaging (Figure 2), the ImageJ software was used to count the number of AuSeeds per particle. Taking into account that TEM images only showed half of the pseudo-spherical NPs, we assumed that the visible AuSeeds belong to that of a hemisphere, and as such, we multiplied the AuSeed count by two to have an estimation of the total number of AuSeeds attached to each LN NP. The sample size for the statistical analysis was 50 LN NPs, from which, the average number of AuSeeds per particle was calculated.

Calculation of average number of AuSeeds per LN NP from ICP-AES results.

The ICP-AES analysis led to the total concentration of Nb and Au atoms in the samples, which allowed deriving the concentrations of LN@BPEI NPs and of AuSeeds, both in units of g/L and NPs/L). From the NPs/L concentration numbers, the amount of LN@BPEI and AuSeeds particles was calculated. Assuming that all the detected gold arises from AuSeeds attached to the LN@BPEI, the average number of AuSeeds per LN particle was then determined.

The mass concentration of LN was calculated using Eq. S3:

$$[LN](g/L) = \frac{[Nb](g/L)}{0.63}$$	(Eq. S3)

where [Nb](g/L) is the mass concentration in Nb obtained from ICP-AES. 0.63 is the mass contribution of Nb based on the empirical formula of $LiNbO_3$.

Then the concentration of LN and AuSeeds in units of NPs per L was determined from Eq. S4 and Eq.S5, respectively:

$$[LN](NPs/L) = \frac{[LN](g/L)}{Mass\ of\ 1\ LN\ NP}$$	(Eq. S4)
$$[AuSeeds](NPs/L) = \frac{[Au](g/L)}{Mass\ of\ 1\ AuSeed}$$	(Eq. S5)

where [Au](g/L) is the mass concentration in Au obtained from ICP-AES. The mass of 1 LN NP as well as the mass of 1 AuSeed was calculated based on the volume and density of a single LN and AuSeed NP, respectively.

Finally, the number of AuSeeds per LN NP, N_{Au} was calculated with Eq. S6:

$$N_{Au} = \frac{[AuSeeds](NPs/L)}{[LN](NPs/L)}$$	(Eq. S6)

Calculation of the percentage of LN surface covered by AuSeeds.

From the number of AuSeeds (diameter: 2.5 nm) per LN NP (diameter: 45nm) and assuming the spherical shape of LN NPs and AuSeeds, the percentage of LN surface covered by AuSeeds can be determined using Eq. S7:

$$\%\ of\ LN\ surface\ covered\ by\ AuSeeds = \frac{N_{Au} \times \pi \times \left(\frac{2.5}{2}\right)^2}{4 \times \pi \times \left(\frac{45}{2}\right)^2}$$	(Eq. S7)

where N_{Au} is the number of AuSeeds per LN NP either determined by TEM or ICP-AES. In Eq. S7, the surface occupied by a single AuSeed on LN surface is supposed to be equal to the area of its circular projection. The ratio between this projection and the area of a LN NP considered as a sphere gives the surface percentage occupied by one AuSeed on LN surface.

Discussion on the limitations of TEM-based quantification compared to ICP-AES analysis.

The linear trend obtained by TEM-based quantification could indicate a potential to further increase the percentage of surface covered by increasing the AuSeeds concentration although a maximum can be expected. This limit is a result of summation effects originating from the electrostatic repulsion between AuSeeds, the Van der Waals attractive forces between the particles and the hydrophobic and steric interactions due to the polymer and solvation forces. Conformation of the BPEI molecules on the LN core also impacts the surface arrangement of NH_2 groups. These factors determine the optimal radial distance between two neighboring

AuSeed particles and prevent full control of the surface spatial arrangement of the seeds. Note also that the AuSeed number is very likely underestimated from the TEM analysis since the statistical approach is first limited by the amount of detected LN@BPEI@AuSeeds. Imaging was indeed performed at high magnification so as to better visualize the surface AuSeeds, which reduced the nanoparticle count per image and consequently the sample size for the statistical analysis. Second, homogeneity in the number of AuSeeds attached to the visible and hemispheres of the LN@BPEI@AuSeeds was assumed since a factor 2 has been used to account for the unseen hemisphere. This approach is however too straightforward. The AuSeeds located at the curved boundaries of the NPs appear blurred preventing in this case their accurate counting. On the contrary, ICP-AES allows obtaining a direct quantification of all the chemical elements present in the sample at a µg/L concentration range. It was thus considered more accurate than the TEM-based quantification.

Expression of the theoretical depolarization ratio *D* for $LiNbO_3$.

Following the initial calculation of the squared hyperpolarizability isotropically averaged over all orientations for a molecular solution,[3] the HRS formalism was then successfully extended to larger objects such as metal NPs [4] and oxide nanocrystals.[5] For $LiNbO_3$, the depolarization ratio obtained after orientational averaging of the HRS intensities collected along the horizontal and vertical directions can be expressed as follows (Eq. S8) according to the three non-zero SHG coefficients d_{33}, d_{31} and d_{22}.

$$D = \frac{I_{HRS}^{H}}{I_{HRS}^{V}} = \frac{\frac{1}{35}d_{33}^{2} + \frac{4}{21}d_{31}^{2} + \frac{16}{105}d_{22}^{2} - \frac{4}{105}d_{33}d_{31}}{\frac{1}{7}d_{33}^{2} + \frac{24}{35}d_{31}^{2} + \frac{8}{35}d_{22}^{2} + \frac{12}{35}d_{33}d_{31}} \quad \text{(Eq. S8)}$$

References

[1] N. Iyi, K. Kitamura, F. Izumi, J. K. Yamamoto, T. Hayashi, H. Asano, S. Kimura, *J. Solid State Chem.* **1992**, *101*, 340.

[2] "Polyethylenimine, branched 408727," Sigma-Aldrich.

[3] S. J. Cyvin, J. E. Rauch, J. C. Decius, *J. Chem. Phys.* **1965**, *43*, 4083.

[4] I. Russier-Antoine, E. Benichou, G. Bachelier, C. Jonin, P. F. Brevet, *J. Phys. Chem. C* **2007**, 9044.

[5] Y. Mugnier, L. Bonacina, in *21st Century Nanosci. - A Handbook, Vol. 4 Low-Dimensional Mater. Morphol.*, Taylor & Francis Group, **2020**.
